# The “Three Italy” of the COVID-19 epidemic and the possible involvement of SARS-CoV-2 in triggering complications other than pneumonia

**DOI:** 10.1007/s13365-020-00862-z

**Published:** 2020-06-16

**Authors:** Carla Prezioso, Maria Elena Marcocci, Anna Teresa Palamara, Giovanna De Chiara, Valeria Pietropaolo

**Affiliations:** 1 Microbiology of Chronic Neuro-degenerative Pathologies, IRCSS San Raffaele Pisana, Rome, Italy; 2 Department of Public Health and Infectious Diseases, “Sapienza” University, P.le Aldo Moro, 5, 00185 Rome, Italy; 3 Department of Public Health and Infectious Diseases, Laboratory affiliated to Istituto Pasteur Italia – Fondazione Cenci Bolognetti, Sapienza University of Rome, Rome, Italy; 4 IRCCS San Raffaele Pisana, Telematic University, Rome, Italy; 5 Institute of Translational Pharmacology, National Research Council, Rome, Italy

**Keywords:** COVID-19, SARS-CoV-2, Italy geographical circulation, Pathophysiology, Cardiovascular manifestations, Neurological implications

## Abstract

Coronavirus disease 2019 (COVID-19), first reported in Wuhan, the capital of Hubei, China, has been associated to a novel coronavirus, the severe acute respiratory syndrome coronavirus 2 (SARS-CoV-2). In March 2020, the World Health Organization declared the SARS-CoV-2 infection a global pandemic. Soon after, the number of cases soared dramatically, spreading across China and worldwide. Italy has had 12,462 confirmed cases according to the Italian National Institute of Health (ISS) as of March 11, and after the “lockdown” of the entire territory, by May 4, 209,254 cases of COVID-19 and 26,892 associated deaths have been reported. We performed a review to describe, in particular, the origin and the diffusion of COVID-19 in Italy, underlying how the geographical circulation has been heterogeneous and the importance of pathophysiology in the involvement of cardiovascular and neurological clinical manifestations.

## Introduction

Coronavirus disease 2019 (COVID-19), a highly infectious disease caused by severe acute respiratory syndrome coronavirus 2 (SARS-CoV-2) was firstly reported in Wuhan, Hubei Province, China, in December 2019 and rapidly spread to other cities and countries beyond China (Zhu et al. 2020). The beginning of the infection started from the Huanan seafood wholesale market, while the exact infection route of the first case remains unclear (Paules et al. 2020). In the early stages of the global COVID-19 spread, the cases identified outside of China were mostly travelers who were infected in China and then moved to areas outside of the country. From mid-February 2020, COVID-19 has begun to spread in South Korea, Italy, Iran, and Japan, and the World Health Organization (WHO) declared this ongoing outbreak as a global public health emergency (WHO Coronavirus disease 2019a). On the March 11, 2020, COVID-19 has been defined as a pandemic (WHO Coronavirus disease 2019b). Currently, SARS-CoV-2 has spread to all continents excluding Antarctica. As of May 11, 2020, a total of 4,006,257 COVID-19 cases with 278,892 deaths were confirmed, among which 44,533 cases were from Africa, 1,702,451 cases were from Americas, 1,731,606 cases were from Europe, 100,881 cases were from South-East Asia, and, finally, 160,910 cases were from Western Pacific (World Health Organization 2020).

This article gives a bird’s eye view about the origin, spread, features, and related emerging complications of COVID-19 disease, focusing on the situation in Italy.

## Origin and spread of COVID-19 in Italy

**Fig. 1:**
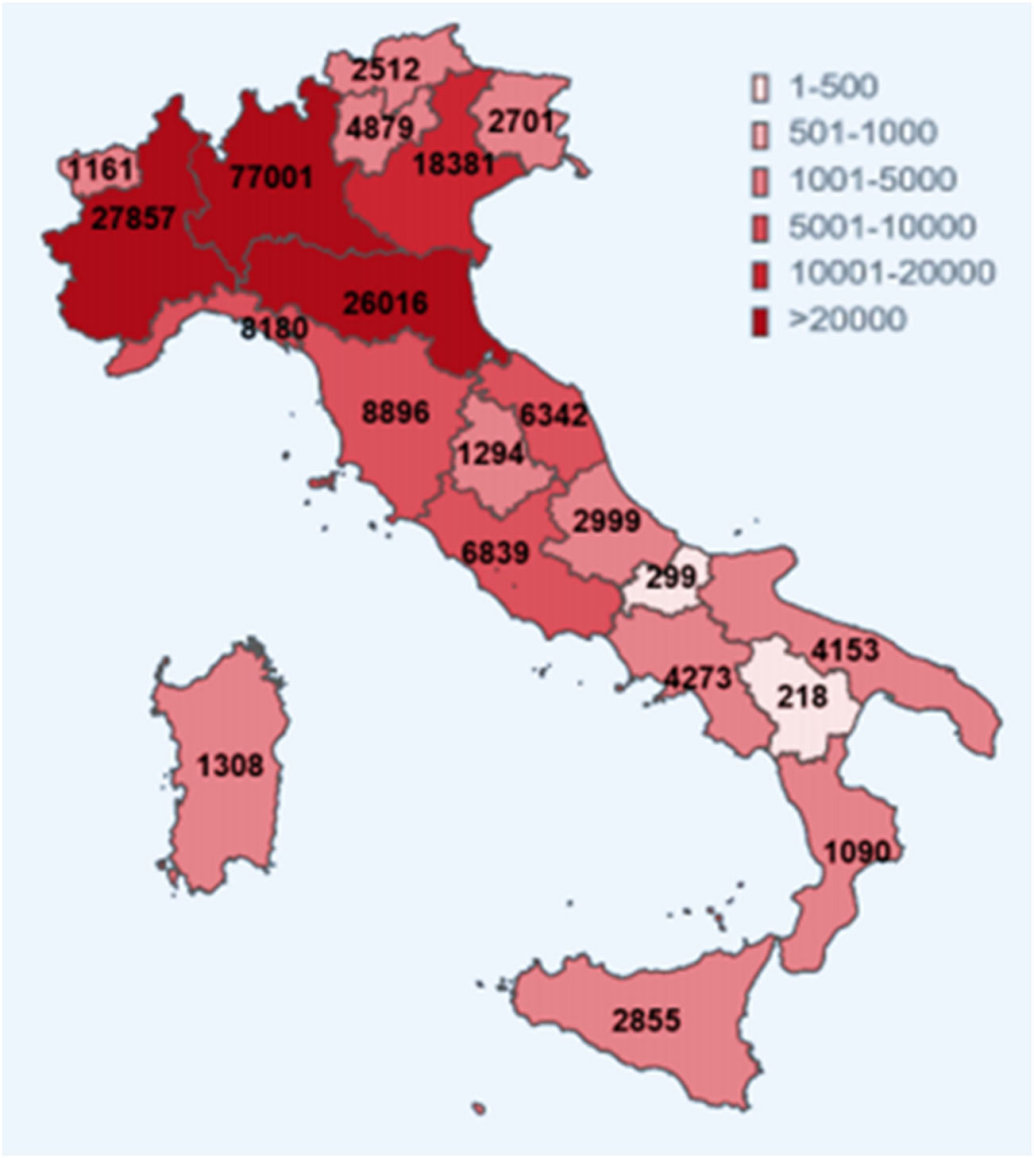
The “Three Italy” of the COVID-19 epidemic. The highest geographical SARS-CoV-2 spread was reported in the northern regions of Italy and the lowest in the southern regions and in the main Islands. The region of Lombardy has the highest number of cases of SARS-CoV-2 and appears to be the epicenter of the Italian outbreak, unlike regions like Molise, Basilicata, and Sardinia, where the SARS-CoV-2 circulation has been encompassed

**Fig. 2:**
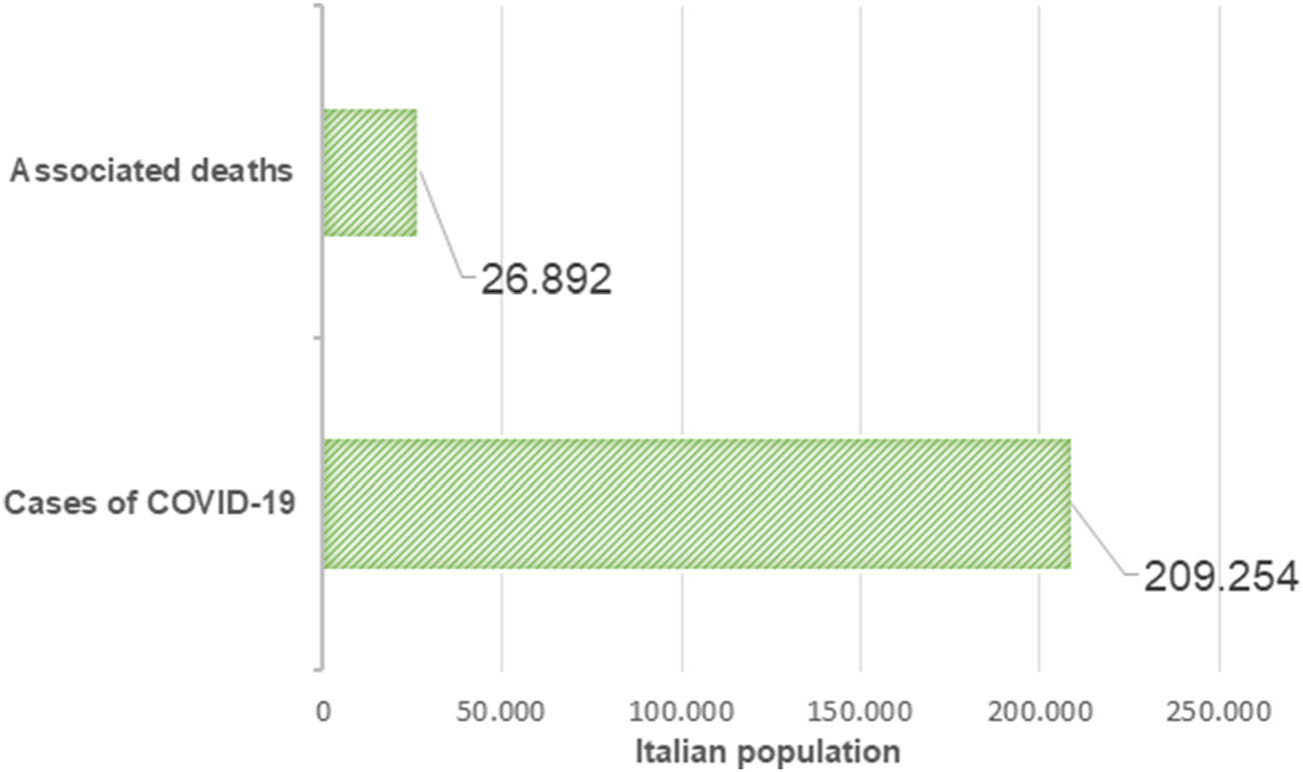
Cases of infected patients and associated deaths. According to the Italian National Institute of Health (ISS), by May 4 in Italy, there were 209,254 cases of COVID-19 and 26,892 associated deaths

**Fig. 3:**
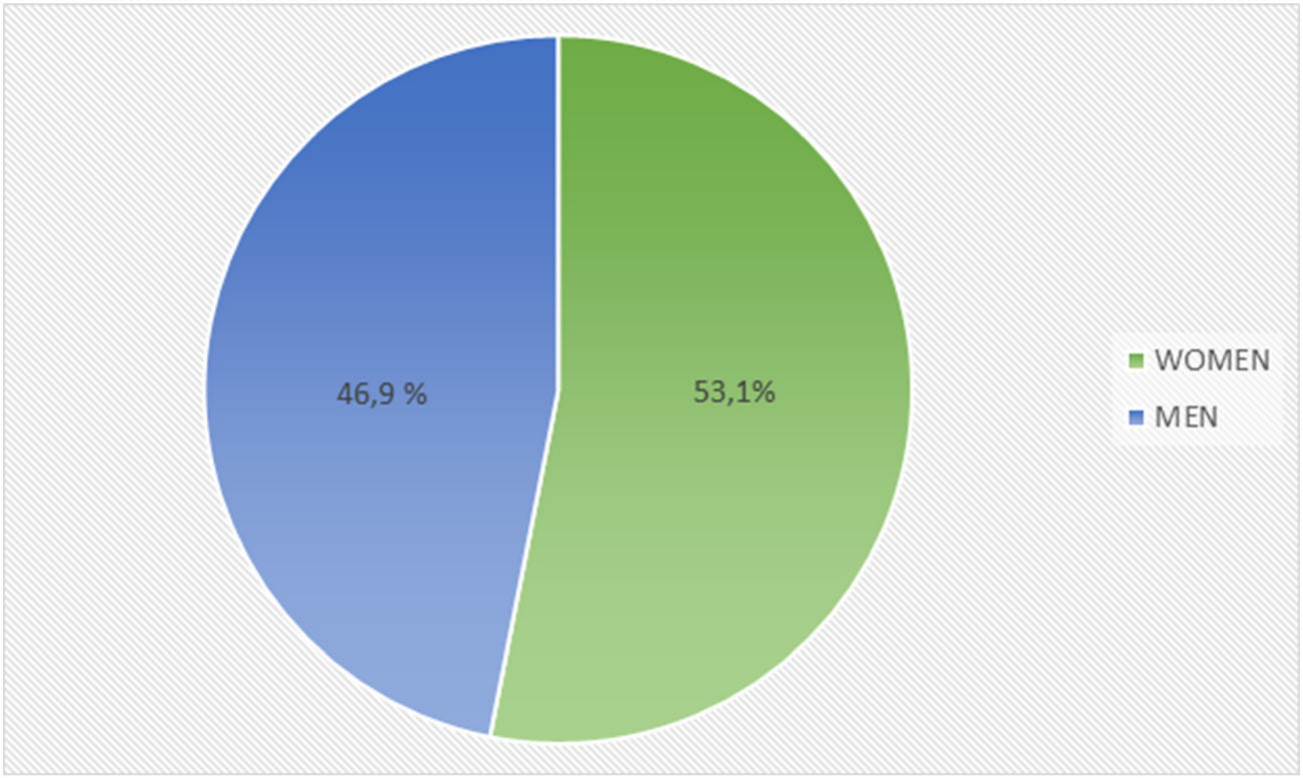
Cases of SARS-CoV-2 infected patients in female and male gender. SARS-CoV-2 was more common among women (53.1%) than among man (46.9%), although lethality is higher in male subjects

The first cases of COVID-19 had been reported in Central Italy, in Rome. The subjects were tourists from Wuhan who had traveled in Lombardy, a Northern region of Italy. The first autochthonous diagnosed case, in the Italian territory, was reported on the February 20, 2020, in a 38-year-old man from the city of Codogno (Lombardy) with no travel history to known areas of viral infection or relation to a probable or confirmed COVID-19 case. After the case in Codogno, other people in Lombardy with flu-like symptoms were found positive for SARS-CoV-2, including medical staff. Thereafter, the Italian COVID-19 epidemic has been mainly characterized by a local transmission. Since the beginning of the epidemic, several preventive measures to favor “social distancing” were undertaken, initially at the local level and finally at the national level with a lockdown of the entire territory on March 11, 2020 (Italian Ministry of Health Covid-19 2020). Geographical spread of the COVID-19 has been heterogeneous with a substantial gradient at the latitude levels with the highest spread in the Northern regions and the lowest in the Southern regions and in the main Islands. The region of Lombardy has the highest number of cases of SARS-CoV-2 and appears to be the epicenter of the Italian outbreak, unlike regions like Molise, Basilicata, and Sardinia, where the SARS-CoV-2 circulation has been encompassed (Italian National Institute of Health (ISS) and National Institute of Statistics 2020) (Fig. 1). According to the Italian National Institute of Health (ISS), by May 4 in Italy, there were 209,254 cases of COVID-19 and 26,892 associated deaths (Italian National Institute of Health (ISS) 2020) (Fig. 2). The last evaluation of the Imperial College COVID-19 Response Team estimated that the infected patients in Italy should be about 9% (95% CI 3.2–26) of the total population (Ferguson et al. 2020). Although cases were more common among women (53.1%), mainly among the elderly population, lethality is higher in male subjects (Fig. 3). The median age at COVID-19 diagnosis was 62 years old. Around 35% of reported COVID-19 cases had at least one diagnosed comorbidity reported: cardiovascular, respiratory, diabetes, immune system deficit, metabolic, cancer, and renal comorbidities (Italian National Institute of Health (ISS) and National Institute of Statistics 2020) SARS-CoV-2 mortality rate (3.8%) (Report of the WHO-China Joint Mission on Coronavirus Disease 2019) is lower than that of other members of Coronaviridae family, such as severe acute respiratory syndrome coronavirus (SARS-CoV) (10%) [World Health Organization (WHO). Summary of probable SARS cases with onset of illness from 1 November 2002 to 31 July. (World Health Organization (WHO) 2020) or Middle East respiratory syndrome coronavirus (MERS-CoV) (37.1%) (WHO Middle East respiratory syndrome coronavirus (MERS-CoV) 2019), although the number of relative infection cases is more than 10 times higher.

Considering only the month of March, the average national level shows an increase in deaths due to the total causes of 49.4%. If we take as a reference the period from the first COVID-19 death reported by the COVID-19 ISS (February 20) until March 31, deaths increase although the strong concentration of the phenomenon in some areas of the country “flatten” the size of the impact of the COVID-19 epidemic on total mortality (Italian National Institute of Health (ISS) 2020)

Specifically, 91% of the excess mortality registered at the average national level in March 2020 is concentrated in the areas of high spread of the epidemic: 37 northern provinces plus Pesaro and Urbino. In all of these provinces, deaths from all causes have more than doubled compared to the 2015–2019 average for the month of March. If we consider the period from February 20 to March 31, 52% are deaths reported by the COVID-19 ISS (Italian National Institute of Health (ISS) 2020)

In the areas with medium spread of epidemic (35 provinces mainly located in the Center-North), death increase due to the total causes in the period from February 20 to March 31 is much more contained: 47% are attributed to COVID-19 positive deaths. Finally, in the areas with a low spread of epidemic (34 provinces mostly located in the Center and in the South), deaths of the month of March 2020 are on average 1.8% lower than that of the last 5 years.

## Transmission, symptoms, and diagnosis

Infection is transmitted from human to human and through contact with contaminated environmental surfaces. Respiratory droplets and contact transmission are considered to be the main transmission routes. Recent reports indicate that SARS-CoV-2 can be detected in the urine and stool of laboratory confirmed patients, implying a risk of fecal–oral transmission (General Office of National Administration of Traditional Chinese Medicine n.d.). Moreover, there is still no evidence that SARS-CoV-2 can be transmitted through aerosols or from mother to baby during pregnancy or childbirth (Yuefei et al. 2020). COVID-19 patients are the main source of infection, and severe patients are considered to be more contagious than mild ones. Asymptomatically infected persons or patients in incubation, without signs or symptoms of respiratory infection, proven to shed infectious virus, may also be potential sources of infection (Bai et al. 2020; Pan et al. 2020; Rothe et al. 2020). These features may explain the sudden epidemic spreading of the virus.

The clinical spectrum of COVID-19 symptoms varies from asymptomatic/pauci-symptomatic forms to clinical conditions, characterized by severe respiratory failure, that necessitates mechanical ventilation and support in an intensive care unit, to multiorgan and systemic manifestations, characterized by sepsis, septic shock, and multiple organ dysfunction syndromes (Lupia et al. 2020). Pneumonia appears to be the most frequent manifestation of infection, with fever, cough, dyspnea, and bilateral infiltrates on chest imaging revealing invasive lesions in both lungs (Yang and Jin, 2020). Other common clinical manifestations included fever, cough, fatigue, sputum production, shortness of breath, sore throat, headache, and olfactory and gustative disorders (Guan et al. 2020a; Guan et al. 2020b). In addition, a part of patients manifested gastrointestinal symptoms, with diarrhea and vomiting, and in some patients, they may be presenting complaint (Chan et al. 2020; Huang et al. 2020). Whether upper respiratory symptoms and gastrointestinal symptoms were rare, fever and cough represented the main symptoms, suggesting the differences in viral tropism as compared with SARS-CoV, MERS-CoV, and influenza virus (Assiri et al. 2013; Lee et al. 2003; Wang et al. 2016).

In severe cases, elderly and subjects with underlying disorders (i.e., hypertension, chronic obstructive pulmonary disease, diabetes, cardiovascular disease) could develop acute respiratory distress syndrome (ARDS), septic shock, metabolic acidosis, and coagulation dysfunction, leading to the death (Huang et al. 2020a). Molecular-based approaches are the first line of methods to confirm SARS-CoV-2-suspected infection. Specifically, reverse-transcription polymerase chain reaction (RT-PCR) is used to detect the nucleic acid of SARS-CoV-2 in sputum, throat swabs, and secretions of the lower respiratory tract samples (Lippi et al. 2020). Although nucleic acid testing is the gold standard technique for laboratory diagnosis, other methods such as virus antigen or serological antibody testing are also valuable assays (Chen et al. 2015; Meyer et al. 2014). A positive test for SARS-CoV-2 generally confirms the diagnosis of COVID-19, although false-positive tests are possible. If initial testing is negative but the suspicion for COVID-19 remains, the WHO recommends resampling and testing from multiple respiratory tract sites(World Health Organization Coronavirus Disease (COVID-19)). Imaging findings of chest computed tomography (CT) in patients with COVID-19 most commonly demonstrate ground-glass opacification with or without consolidative abnormalities, consistent with viral pneumonia. Other studies have suggested that chest CT abnormalities are more likely to be bilateral, have a peripheral distribution, and involve the lower lobes. Chest CT may be helpful in making the diagnosis, but no finding can completely rule in or rule out the possibility of COVID-19. Negative RT-PCR tests on oropharyngeal swabs despite CT findings suggestive of viral pneumonia have been reported in some patients who ultimately tested positive for SARS-CoV-2. Serologic tests should be able to identify patients who have either current or previous infection but a negative PCR test (Lim et al. 2020; Ling et al. 2020). Previous studies have demonstrated that the development of antibody response to infection can be host dependent and take time; in the case of SARS-CoV-2, the majority of patients seroconvert between 7 and 11 days post-exposure to the virus, although some patients may develop antibodies sooner (Patel et al. 2020). As a result of this natural delay, antibody-detecting tests for SARS-CoV-2 are not useful for the diagnosis of an acute illness but might be important for understanding the diffusion of Sars-CoV-2 in symptomatic or pauci-symptomatic patients.

## Virus and pathophysiology

SARS-CoV-2, taxonomically, has been classified as *Betacoronavirus* (lineage B) by the WHO (Zhu et al. 2020). Genetic sequence analysis showed more than 50% homology to MERS-CoV and 80% to SARS-CoV and both originate in bats. The differences between the sequences are mainly in the ORF1a gene and the spike gene, encoding S-protein, which is the key protein for the interaction between coronavirus and host cells (Lu et al. 2020; Zhou et al. 2020; Zhu et al. 2020). The coronaviruses (CoVs) family is a class of enveloped, positive-sense single-stranded RNA viruses having an extensive range of natural roots. These viruses can cause respiratory, enteric, hepatic, and cardiovascular diseases (de Wilde et al. 2018; Weiss and Leibowitz 2011; Zheng et al. 2020). SARS-CoV-2 virion presents a genome size of 29.9 kb [Available via http://nmdc.cn/coronavirus] with a nucleocapsid composed of genomic RNA and phosphorylated nucleocapsid (N) protein. The nucleocapsid is covered by an envelope with the spike (S) glycoprotein trimmer, which exists in all CoVs, and the hemagglutinin-esterase (HE), only expressed from some CoVs. Moreover, in the viral envelope, located among the S protein, are confined the membrane (M) and the envelope (E) proteins (Wu et al. 2020). The SARS-CoV-2 genome contains a variable number of open reading frames (ORFs) (Song et al. 2019) encoding for structural proteins, including S, M, E, N proteins and accessory proteic chains (Lei et al. 2018; Letko et al. 2020). To address the pathogenetic and virulence mechanisms of SARS-CoV-2, the role of structural and non-structural proteins (nsps) must be considered (Letko et al. 2020). Four structural proteins are essential for virion assembly and infection of CoVs (Di Gennaro et al. 2020). S protein, present on the viral surface as a trimer, is the primary determinant of viral tropism and is responsible for receptor binding and membrane fusion (Beniac et al. 2006; Delmas and Laude 1990). The M protein has three transmembrane domains. It shapes the virions, promotes the curvature of the membrane, and binds to the nucleocapsid (Nal et al., 2005; Neuman et al. 2011). Regarding the E protein, it plays a role in virus assembly, release and viral pathogenesis (DeDiego et al. 2007; Nieto-Torres et al. 2014). Finally, the N protein contains two domains, both of which can bind virus RNA genome via different mechanisms. It is reported that N protein can bind to nsp3 protein packaging the encapsidated genome into virions (Chang et al. 2006; Fehr and Perlman, 2015; Hurst et al. 2009). Moreover, N is also an antagonist of interferon and viral encoded repressor of RNA interference, which appears to be beneficial for the viral replication (Cui et al. 2015). Among nsps, most of these proteins, specifically nsp1 to 16, displayed a specific role in CoVs replication and in blocking the host innate immune response (Cascella et al. 2020; Guo et al. 2020). The pathogenic mechanism that starts with SARS-CoV-2 infection and culminates in pneumonia and heart or other extensive tissue damage seems to be particularly complex and able to produce an overreaction of the immune system associated with a pronounced cytokine storm, also known as cytokine release syndrome (CRS). Clinical features include extremely elevated cytokine levels (IL-6, IL-10, and TNF-α), lymphopenia (in CD4+ and CD8+ T cells), decreased IFN-γ expression in CD4+ T cells, and an increase in Th17 cell proportion. Th17 cells are helper T cells differentiated from Th0 cells mainly stimulated by IL-6 and IL-23. Specifically, IL-6, an important member of the cytokine network and produced by activated macrophages, plays a central role in acute inflammation with its anti-inflammatory and pro-inflammatory effects. Biologically, IL-6 promotes T cell population proliferation and activation and B cell differentiation, regulates acute phase response, and affects the hormone-like properties of vascular disease, lipid metabolism, insulin resistance, mitochondrial activity, neuroendocrine system, and neuropsychological behavior (Chen et al. 2020a; Pyle et al. 2017). On the other end, IL-6 increases during inflammatory and cardiovascular diseases (myocardial ischemia, coronary atherosclerosis, angina pectoris, congestive heart failure, hypertension), infections, autoimmune disorders, and some types of cancer (Bennardo et al. 2020). During the spread of infection, SARS-CoV-2 could cross through the mucous membranes, especially nasal and larynx mucosa; enters the lungs through the respiratory tract (Bennardo et al. 2020; Rose-John 2018; Chen et al. 2020b); and could cause the aggravation of patient’s condition in 7 to 14 days after onset. B lymphocyte reduction may occur early during the disease and may affect antibody production in patients. Elevated IL-6 levels were observed in patients infected by SARS-CoV-2 and were correlated with the exacerbation of the disease around 2 to 10 days after onset (Di Gennaro et al. 2020). Excessive IL-6 signaling leads to a many of biological effects, such as maturing naïve T cells into effector T cells, the induction of vascular endothelial growth factor (VEGF) expression in epithelial cells, the increase of vessel permeability, and reducing myocardium contractility, culminating in organ damage (Pathan et al. 2004; Tanaka et al. 2016). Since elevated IL-6 levels were consistently reported during COVID-19 and were correlated with higher mortality in these patients, IL-6 might serve as a predictive biomarker for COVID-19 severity.

## The role of angiotensin-converting enzyme 2 (ACE2)

**Fig. 4:**
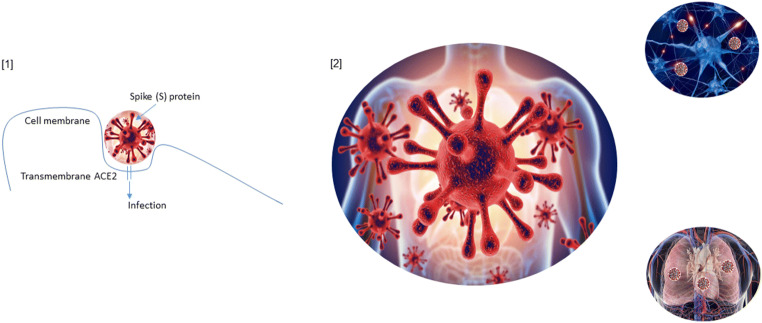
Infection mechanism and tissue distribution of ACE2 receptors in humans. **1** The mechanism of the SARS-CoV-2 intracellular entry involves the viral Spike (S) protein C-terminal domain containing a receptor-binding region that binds to the extracellular domain of angiotensin-converting enzyme 2 (ACE2). Cleavage of the S protein by the host transmembrane serine protease 2 (TMPRSS2) is a crucial step for the membrane fusion and viral internalization by endocytosis with ACE2. **2** Different organs participate in COVID-19 due to the wide range expression of the primary SARS-CoV-2 entry receptor ACE2. ACE2 is particularly expressed on the type II alveolar epithelial cells, heart, and brain. The different distribution of ACE2 in organs and tissue is significantly correlated to the clinical symptoms of SARS-CoV-2 infection

Different organ systems are believed to participate in COVID-19 due to the wide range expression of the primary SARS-CoV-2 entry receptor, angiotensin-converting enzyme 2 (ACE2) (Fig. 4).

ACE2 is particularly expressed on the type II alveolar epithelial cells (Guan et al. 2020a; Guan et al. 2020b; Wang and Xu 2020; Yang et al. 2020a; Yang et al. 2020b; Yang et al. 2020c; Zhao et al. 2020) although also the gastrointestinal tract, kidneys, heart, brain stem, vasculature, liver, and nasal and oral mucosa express these receptors, contributing to extra-pulmonary manifestations (Zhao et al. 2020). However, the different distribution of ACE2 in organs and tissue is significantly correlated to the clinical symptoms of SARS-CoV-2 infection (Nejadi Babadaei et al. 2020) (Fig. 4).

In addition to functional receptor role, physiologically, ACE2 is an important modulator of the renin-angiotensin-aldosterone system (RAAS), which systemically regulates, via distinct hormones, the cardiovascular and immune systems and is involved in heart function and in the development of hypertension and diabetes mellitus. Briefly, the pro-renin is cleaved in the kidneys, and then, the activated renin is released into the blood stream where it in turn activates angiotensinogen, which is converted into angiotensin I and finally into angiotensin II (AngII). Specific angiotensin-converting enzymes (ACEs), mainly expressed on renal and pulmonary epithelium, facilitate this activation cascade. ACE2 is a well-characterized negative regulator of the RAAS system as it converts AngII into the vasodilatory fragment Ang 1–7, which simultaneously decreases the AngII concentration to facilitate the antihypertensive effect (Keidar et al. 2007; Santos et al. 2018).

## COVID-19 and cardiovascular manifestations

ACE2, located also on the cell surface of cardiac systems, may help to understand those patients infected by SARS-CoV-2 that presented cardiovascular symptoms (myocardial injury, cardiac chest pain, fulminant heart failure, cardiac arrhythmias, and cardiac death), in addition to the typical respiratory symptoms during COVID-19 (Long et al. 2020) (Fig. 4). The mechanism for the SARS-CoV-2 intracellular entry involves the viral S protein C-terminal domain containing a receptor-binding region that binds to the extracellular domain of ACE2 (Di Gennaro et al. 2020). Cleavage of the S protein by the host transmembrane serine protease 2 (TMPRSS2), to generate the S1 and S2 subunits, is a crucial step for the membrane fusion and viral internalization by endocytosis with ACE2 (Hoffmann et al. 2020) (Fig. 4). Interestingly, the interaction between S protein with the extracellular domain of ACE2, which triggers the endocytosis of the complex, is remarkably increased in patients suffering from hypertension or coronary heart disease (Svenningsen et al. 2017; Zheng et al. 2016). ACE2 internalization by SARS-CoV-2 results in the loss of ACE2 at the cell surface and removes a key pathway for the cell to degrade ANG II and generate the cardiovascular-protective Ang-(1–7). Certainly, an increase in the overall ratio of ANG II/Ang-(1–7), following ACE2 internalization, may exacerbate the pulmonary tissue damage, initially caused by SARS-CoV-2 (Guan et al. 2020a; Guan et al. 2020b; Huang et al. 2020; Yang et al. 2020a; Yang et al. 2020b; Yang et al. 2020c). Moreover, the down-regulation of ACE2 on the surface of infected cells might secondary imply an excessive response of the immune system and, then, the progression and the worsening of the disease in SARS-CoV-2-infected patients. Lung and heart damage is due precisely by an overreaction of the immune system associated with a pronounced cytokine storm resulting into vascular inflammation, plaque instability, myocardial inflammation, and hypercoagulable state [(Levi et al. 2004; Prabhu 2004); Yang et al. 2020]. Clinical features include the dysregulation of T helper cells of type 1 and 2 and levels extremely elevated of IL-6 and ferritin (Liu et al. 2020; Yang et al. 2020a; Yang et al. 2020b; Yang et al. 2020c), which are also well-known markers for sepsis. The cardiovascular system involvement, during severe COVID-19, is unquestioned although the specific mechanisms remain to be explored. One proposed mode of action is related to a direct infection of cardiomyocytes (CMCs) by SARS-CoV-2 during the critical phase that leads to the myocarditis development (Zheng et al. 2020). A second assumption is that an over-activated immune system is predominantly contributing to SARS-CoV-2-induced heart damage. Following to cardiac ischemia as an effect of pneumonia and ARDS (Chen et al. 2020a; Chen et al. 2020b; Guan et al. 2020a; Guan et al. 2020b), CMC damage induces a range of pro-inflammatory mechanisms, which in turn escape control and further promote loss of CMCs (Mehta et al. 2020). Consequently, a secondary induced cardiac injury seems to be plausible. However, this scenario is strongly supported by previous studies investigating the SARS-CoV outbreak in 2002 or MERS-CoV-associated epidemic in 2012 (World Health Organization (WHO) 2020), where acute myocarditis and heart failure have also been reported (Alhogbani 2016). Considering the multiple effects on the heart, it can be assumed that previous cardiovascular diseases (CVDs) could lead to worse progression of COVID-19 and to an increased risk of mortality (Long et al. 2020). Interestingly, the increased mortality rate in patients with preexisting or new cardiac injuries is concomitant with high levels of troponin T (TnT) serum, a common cardiac damage biomarker. To support this hypothesis, the study of Guo *and colleagues* reported that CVD patients, without significantly raised TnT serum levels, presented only a slightly increased mortality compared to patients without CVDs (Guo et al. 2020). Therefore, although TnT levels should always be monitored in the case of cardiac damage caused by comorbidities (hypertension, diabetes, cardiovascular but also pulmonary dysfunctions, cancer, nephrological and cerebrovascular disorders) or severe disease progression, these levels should be critically interpreted since some medications and interventions can cause an upregulation of TnT (Cardiology Magazine 2020; Harvell et al. 2016). Together with the inflammatory cytokine IL-6 and ferritin, TnT may be used to predict the outcome of the SARS-CoV-2 infection and help to reduce COVID-19 mortality (Poe et al. 2015; Rivara et al. 2012).

## Neurological implications

A growing body of clinical findings suggest that SARS-CoV-2 gain entry into the central nervous system (CNS) (Mao et al. 2020), but the exact route of access has yet to be demonstrated. According to similarity with other coronaviruses, including MERS-CoV and SARS-CoV and studies in animal model of infection (Netland et al. 2008), CNS invasion was postulated to occur mainly by trans-synaptic transfer of virus particles, starting from infection in the peripheral nerve endings such as the olfactory ones (Butowt and Bilinska 2020). Along this line, peripheral neuronal manifestations, as the loss of smell and taste, are frequently experienced by COVID-19 individuals (Baig et al. 2020; Mao et al. 2020). The expression of ACE2 on the surface on neurons and glial cells (Hamming et al. 2004) (Fig. 2) as well as the priming proteases TMPRSS2 and furin, all required for virus attach and entry in host cells (Hoffmann et al. 2020; Lashley et al. 2008) makes them suitable targets for virus infection. However, recent findings from a postmortem study (Paniz-Mondolfi et al. 2020) support the hypothesis that the virus could also exploit the hematogenous route, since SARS-CoV-2 was found in neurons and capillary endothelial cells of the frontal lobe of a patient death for SARS-CoV2-associated ARDS. It is known that endothelial cells of blood vessels, including the cerebral ones, express ACE2. Thus, SARS-CoV-2 could infect those cells in the cerebral blood vessels, leading to increased permeability of the blood brain barrier (BBB) and in turn promoting pathogen invasion into the brain. A similar effect may be exerted indirectly by the virus-induced inflammatory cytokine storms, leading to brain edema and intracranial hypertension. Finally, the virus might also exploit a macrophage-mediated Trojan-horse mechanism (Santiago-Tirado and Doering 2017) or the lymphatic route to reach the brain, as well as other organs.

Several COVID-19 patients showed secondary mild neurological symptoms, including headache, dizziness, and neuralgia, whereas some severe cases showed neurological complications such as encephalopathy and acute cerebrovascular diseases (Ahmad and Rathore 2020). Along this context, besides other primary neurological manifestations such as seizures, increasing evidence indicate that SARS-CoV-2 can cause meningitis and encephalitis. Several reports documented encephalitis without cerebrospinal fluid (CSF) virus detection in COVID-19 patients (Bernard-Valnet et al. 2020; Dogan et al. 2020; Duong et al. 2020; Ye et al. 2020). Poyiadji *and colleagues* reported the first US presumptive case of COVID-19-associated acute hemorrhagic necrotizing encephalopathy (Poyiadji et al. 2020), a rare encephalopathy previously associated with Influenza A virus (IAV) and potentially related to damaging effects of the virus-induced cytokine storm on BBB and brain parenchyma (Bailey 2020). The MRI brain of this SARS-CoV-2-positive 50-year-old female patient with a 3-day history of cough, fever, and altered mental status showed hemorrhagic rim resulting in lesions within the bilateral thalami, medial temporal lobes, and sub-insular brain regions. CSF samples resulted negative for bacteria and herpesviruses, but no investigation was performed either for IAV or SARS-CoV-2 presence. Bernard-Valnet *and colleagues* described two patients who developed a meningoencephalitis few days after a diagnosis of SARS-CoV-2 infection. At first, their symptoms were the classic ones of COVID-19 patients, including mild respiratory symptoms. Then, they developed severe neuropsychological symptoms, compatible with viral meningoencephalitis. However, no viruses, including SARS-CoV-2, were detected in their CSF samples (Bernard-Valnet et al. 2020). Another case was reported in Iran, where a hospitalized 79-year-old male, with history of fever and cough and positive for SARS-CoV-2, entered in a semi-conscious state probably due to a massive intracerebral hemorrhage in the right hemisphere with intraventricular and subarachnoid extension, as revealed with computed tomography scan. The authors did not report results from CSF analysis, but just hypothesized that SARS-CoV-2 may directly invades the CNS via the olfactory receptors of cranial nerve I in the nasal cavity cell membrane (Sharifi-Razavi et al. 2020). Dogan *and colleagues* documented 6 cases of SARS-COV2-related autoimmune meningoencephalitis among the 29 most severely affected COVID-19 patients (intubated) of the 332 ones admitted to their center. Despite CSF samples did not show the occurrence of active CNS infection, including SARS-CoV-2, clinical tests evidenced increased levels of acute-inflammation (high ferritin, fibrinogen, CRP, and IL-6 in sera) and bilateral cerebral inflammation compatible with meningoencephalitis on MRI (Dogan et al. 2020).

Very recently, Pilotto *and colleagues* described the case of a SARS-CoV-2-positive 60-year-old Italian patient with a transient akinetic mutism due to encephalitis. The analysis of CSF, negative for several neurotropic viruses such as herpes (HSV-1 and 2, HHV-6, HHV-8, EBV, VZV), adenovirus, enterovirus, and Sars-CoV-2, revealed the high presence of pro-inflammatory cytokines, such as IL-6, TNF-α, IL-8, and pleocytosis, suggesting a possible cytokine-mediated hyperinflammation response to SARS-COV2 infection (Pilotto et al. 2020). Interestingly, additional cases have been supported by positive virus detection in CSF. Morigughi *and colleagues* described the occurrence of meningoencephalitis (diagnosis of aseptic encephalitis) in a 24-year-old male patient with SARS-CoV2 RNA positive detection in CSF samples, but not in nasopharyngeal swab. This man was found unconsciousness and with a stiff neck 9 days after symptom onset (headache, fever, and generalized fatigue at day 1, treated with antiviral and antipyretics under a first diagnosis of influenza; additional symptoms were worsening headache and sore throat at day 5) and experienced a transient generalized seizure during emergency transport to the hospital, and multiple epileptic seizures during emergency hospitalization. He had high CSF pressure (320mmH_2_O) and no evidence of brain edema. Further brain MRI revealed right lateral ventriculitis and encephalitis mainly on right mesial lobe and hippocampus and pan-paranasal sinusitis. Authors claimed that the symptoms of encephalitis and cerebropathia should be considered as first indication, as the respiratory ones, for the diagnosis of hidden COVID-19 patients, and suggest the importance to pay attention to nasal and paranasal condition in the diagnosis and treatment for SARS-CoV-2 (Moriguchi et al. 2020).

Huang *and colleagues*, by updating what described by Duong *and colleagues* (Duong et al. 2020), also reported a case of SARS-CoV2 encephalitis (first CSF analysis negative for herpesviruses and other microbes, subsequent CSF analysis positive for SARS-COV2) in a US 41-year-old woman with some comorbidities (type 2 diabetes mellitus and obesity) whose nasopharyngeal swab on hospital admission was positive for SARS-CoV-2 and negative for influenza A and B viruses. This woman presented fever, headache, and new seizure onset and experienced no respiratory distress nor potential virus-induced abnormalities in other organs. Neurologically, she had hallucination and disorientation, followed by lethargic status but gradually improved after hydrochlorine treatment up to showing no neurologic symptoms within by day 12 of hospitalization (Huang et al. 2020b).

## Kawasaki outbreaks during COVID-19

Although clinical manifestations of COVID-19 children were generally less severe than those of adult patients, young children, particularly infants, were vulnerable to SARS-CoV-2 infection. In children, the respiratory involvement appears to have a more benign course, with almost no fatalities reported in this age group (Dong et al. 2020; Nicastro et al. 2020; Yonker et al. 2020).

However, as observed in adults, also in children, the respiratory tract seems not to be the only system susceptible to SARS-CoV-2 infection (Xiao et al. 2020), and the tissue damage during COVID-19 is mostly mediated by the host innate immunity and by a cytokine storm (D’Antiga 2020; Henderson et al. 2020; Mehta et al. 2020). Kawasaki disease is an acute and typically self-limiting vasculitis, which almost exclusively interest children (Kawasaki et al. 1974). Patients with Kawasaki disease, during the acute phase, might have instability at hemodynamic level, a condition known as Kawasaki disease shock syndrome (KDSS) (Kanegaye et al. 2009). Other patients with Kawasaki disease might present macrophage activation syndrome (MAS) (Wang et al. 2015). The cause of Kawasaki disease remains unknown although the most accredited hypothesis supports an aberrant response of the immune system to one or more unidentified pathogens in genetically predisposed patients (Rowley 2018; Shulman and Rowley 2015). In Japan, during three epidemics in 1979, 1982, and 1986, the highest Kawasaki disease incidence was reported in January, suggesting that factors, during winter months, could trigger Kawasaki disease. During the SARS-CoV-2 pandemic in Italy, as reported by Verdoni *and colleagues*, a high number of Kawasaki-like disease cases (features of patients with Kawasaki disease diagnosed during the COVID-19 pandemic appeared to differ from canonical cohort of Kawasaki patients) occurred after the first case of COVID-19 diagnosed in Bergamo, with a monthly incidence at least 30 times greater than the monthly incidence of the previous 5 years (Verdoni et al. 2020).

In the past 20 years, it has been proposed that viruses of the CoVs family may be implicated in the pathogenesis of Kawasaki disease. In 2005, the New Haven coronavirus (HCoV-NH) has been found in the respiratory secretions of 8 of 11 children with Kawasaki disease versus 22 controls tested (Esper et al. 2005). Skepticism on this association was expressed by a group from Japan, who did a retrospective study on nasopharyngeal swabs from 19 children with Kawasaki disease and 208 controls with respiratory tract infections. HCoV-NH RNA was found only in 5 of 208 controls versus 0 of 19 children with Kawasaki disease (Ebihara et al. 2005; Esper et al. 2005; Turnier et al. 2015). Another Japanese group explored, by serological tests, the association between HCoV-NL63 and HCoV-229E and Kawasaki disease. No difference in HCoV-NL63 antibody positivity between patients and controls was detected, whereas a higher HCoV-229E antibody positivity was reported in patients with Kawasaki disease (Shirato et al. 2014). This data suggests that the CoVs family could represent one of the triggers of Kawasaki disease. In the study of Verdoni *and colleagues*, the presence of the virus was confirmed by the antibodies research versus SARS-CoV-2 in 8 of 10 patients affected by Kawasaki disease. In the remaining two patients, the researchers speculated that confounding factors, such as the serological test performed after an infusion of high-dose immunoglobulins, played a role (Verdoni et al. 2020). Only 2 of 10 patients presented a naso-oropharyngeal swabs positive for SARS-CoV-2 RNA and the positivity of IgG antibodies. These findings suggest a late onset of the disease compared with the primary infection. This could be the reason why, in the past, no active viral infection could be demonstrated in this disease (Verdoni et al. 2020). All these results and considerations could support the hypothesis that the immune response to SARS-CoV-2 is responsible for a Kawasaki-like disease in susceptible patients. Since patients diagnosed with Kawasaki-like disease after the viral spreading revealed a severe course, including KDSS and MAS, requiring adjunctive steroid treatment, genetic studies, in larger groups, should be done in order to investigate the susceptibility of patients developing this disease to the triggering effect of SARS-CoV-2.

## Data Availability

No datasets were generated during the study.

## Abbreviations

COVID-19: Coronavirus disease 2019
SARS-CoV-2: Severe acute respiratory syndrome coronavirus 2
ISS: Italian National Institute of Health
WHO: World Health Organization
SARS-CoV: Severe acute respiratory syndrome coronavirus
MERS-CoV: Middle East respiratory syndrome coronavirus
ARDS: Acute respiratory distress syndrome
RT-PCR: Reverse-transcription polymerase chain reaction
CT: Chest computed tomography
CoVs: Coronaviruses
N: Phosphorylated nucleocapsid
S: Spike
HE: Hemagglutinin-esterase
M: Membrane
E: Envelope
ORFs: Open reading frames
nsps: Non-structural proteins
CRS: Cytokine release syndrome
VEGF: Vascular endothelial growth factor
ACE2: Angiotensin-converting enzyme 2
RAAS: Renin-angiotensin-aldosterone system
AngII: Angiotensin II
ACEs: Specific angiotensin-converting enzymes
TMPRSS2: Transmembrane serine protease 2
CMCs: Cardiomyocytes
CVDs: Cardiovascular diseases
TnT: Troponin T
CNS: Central nervous system
BBB: Blood-brain barrier
CSF: Cerebrospinal fluid
IAV: Influenza A virus
KDSS: Kawasaki disease shock syndrome
MAS: Macrophage activation syndrome
HCoV-NH: New Haven coronavirus
